# A structurally heterogeneous transition state underlies coupled binding and folding of disordered proteins

**DOI:** 10.1074/jbc.RA118.005854

**Published:** 2018-12-04

**Authors:** Elin Karlsson, Eva Andersson, Jakob Dogan, Stefano Gianni, Per Jemth, Carlo Camilloni

**Affiliations:** From the ‡ Department of Medical Biochemistry and Microbiology, Uppsala University, SE-75123 Uppsala, Sweden,; the § Department of Biochemistry and Biophysics, Stockholm University, SE-10691 Stockholm, Sweden,; the ¶ Istituto Pasteur-Fondazione Cenci Bolognetti and Istituto di Biologia e Patologia Molecolari del CNR, Dipartimento di Scienze Biochimiche “A. Rossi Fanelli,” Sapienza Università di Roma, 00185 Rome, Italy, and; the ‖ Dipartimento di Bioscienze, Università degli Studi di Milano, 20133 Milano, Italy

**Keywords:** intrinsically disordered protein, pre-steady-state kinetics, protein folding, protein–protein interaction, molecular dynamics

## Abstract

Many intrinsically disordered proteins (IDPs) attain a well-defined structure in a coupled folding and binding reaction with another protein. Such reactions may involve early to late formation of different native structural regions along the reaction pathway. To obtain insights into the transition state for a coupled binding and folding reaction, we performed restrained molecular dynamics simulations using previously determined experimental binding Φ_b_ values of the interaction between two IDP domains: the activation domain from the p160 transcriptional co-activator for thyroid hormone and retinoid receptors (ACTR) and the nuclear co-activator binding domain (NCBD) of CREB-binding protein, each forming three well-defined α-helices upon binding. These simulations revealed that both proteins are largely disordered in the transition state for complex formation, except for two helices, one from each domain, that display a native-like structure. The overall transition state structure was extended and largely dynamic with many weakly populated contacts. To test the transition state model, we combined site-directed mutagenesis with kinetic experiments, yielding results consistent with overall diffuse interactions and formation of native intramolecular interactions in the third NCBD helix during the binding reaction. Our findings support the view that the transition state and, by inference, any encounter complex in coupled binding and folding reactions are structurally heterogeneous and largely independent of specific interactions. Furthermore, experimental Φ_b_ values and Brønsted plots suggested that the transition state is globally robust with respect to most mutations but can display more native-like features for some highly destabilizing mutations, possibly because of Hammond behavior or ground-state effects.

## Introduction

Protein–protein interactions involving intrinsically disordered proteins (IDPs)^3^ are pivotal in cellular signaling pathways. The flexibility of the IDP allows for extended binding surfaces for recognition and easy access for kinases and phosphatases that modulate the affinity of the protein–protein interaction by (de)phosphorylation. Another aspect of protein–protein interactions of IDPs is that the binding often involves (partial) folding of the IDP into a well-defined structure in the protein–protein complex, a process that in itself can modulate the affinity of the interaction. The details of such coupled binding and folding reactions have therefore recently been addressed to better understand how the disorder of the interacting proteins is affecting the mechanism of the interaction and how it may provide functional advantages (1, 2). The emerging picture is that coupled binding and folding reactions, much like protein folding, can employ mechanisms ranging from a largely nonspecific initial encounter (corresponding to pure nucleation–condensation in protein folding) (3–6) to substantial preformed structure (corresponding to diffusion–collision in protein folding) (7–9). Most studies suggest that the initial stages of a protein–protein interaction, in which at least one binding partner is an IDP, contain only a very small fraction of “native” interactions, *i.e.* interactions that are present in the bimolecular complex at equilibrium. However, there is one example of a highly structured α helix in the transition state (TS) for coupled binding and folding of cMyb to KIX (10).

We have previously performed extensive mechanistic studies on one of the classical examples of a coupled binding and folding reaction, that between ACTR and NCBD (11). The kinetics are complex, reflecting accumulation of various intermediates depending on the conditions. First, NCBD, which was described as a molten globule–like protein domain (12), displays ground state heterogeneity on a submillisecond time scale (13, 14) and on a time scale over tens of seconds related to a proline *cis-trans*-isomerization (15). Second, the binding between NCBD and the highly disordered ACTR involves two concentration-dependent kinetic phases related to the initial steps of association: one detected in stopped-flow experiments that is the major binding phase (16) and one detected in single molecule experiments involving the *cis*-proline conformation of NCBD (15). Third, in stopped-flow binding experiments, two additional phases were observed (τ ∼ 25 ms and τ ∼ 0.9 s, respectively, at 4 °C). The 25-ms phase is observed only in presence of trimethylamine *N*-oxide (TMAO) or high salt. The bases for both of these phases are likely structural rearrangements following the initial binding event. Fourth, in single molecule experiments, a submillisecond phase was detected following initial binding that might be related to a high-energy intermediate (17). Our experimental studies on this interaction involved site-directed mutagenesis and kinetic experiments to obtain Φ_b_ values reporting on the degree of native contacts formed in the TS for the initial binding event. The implications of the complex binding kinetics for interpretation of Φ_b_ values were discussed in detail (18). It was shown that Φ_b_ values will report on the initial binding event for the major binding pathway, and this holds true for the *cis-trans*-proline heterogeneity in NCBD as well, where the major *trans*-proline NCBD conformation will dominate the observed kinetic phase in stopped-flow experiments. The Φ_b_ analysis suggested that most native hydrophobic interactions form late in the reaction, downhill of the TS for binding (18), whereas helix 1 in ACTR is partially formed at the top of the barrier (19). By inference, several initial and TS interactions along the reaction pathway are non-native, *i.e.* not found in the complex at equilibrium. In the present work we use molecular dynamics (MD) simulations restrained by the previous Φ_b_ values, followed by additional experiments and reanalysis of previous double-mutant data to obtain a complete picture of the binding TS of this paradigmatic coupled binding and folding reaction.

## Results

We have here obtained a structural model of the TS for the coupled binding and folding reaction of NCBD and ACTR by a three-step approach. First we used restrained MD simulations (20–22) using previously determined experimental Φ_b_ values based on single-point mutations (18, 19). Hereby we obtained a structural ensemble for the binding TS. Second, we made new single-point mutants to test the MD simulations. Third, to map the plasticity of the TS, we used linear free energy diagrams to reanalyze another previously published kinetic data set on the ACTR–NCBD interaction that was based on double mutants. Briefly, the binding kinetics of all single mutants of ACTR were measured with all single mutants of NCBD. This will probe how sensitive the second mutation is to perturbation by the first mutation.

### Φ_b_ restrained MD simulations of the ACTR–NCBD interaction

To obtain a structural ensemble of the TS, MD simulations were run in GROMACS using the Amber03w force field as detailed under “Experimental procedures.” We included Φ_b_ values in the 0–1 range from single-point mutations (18, 19) to restrain the MD simulation (Table S1). The reason for using the 0–1 range is that the standard interpretation of a Φ value is in terms of fraction of native contact. Most of the included values were conservative deletion mutations. However, three substitutions in helix 1 of ACTR involved polar or charged groups, which complicates interpretation of Φ values caused by solvation effects, which do not cancel out (23). However, these mutations were designed to specifically probe secondary structure propensity, and this effect was confirmed by NMR (19). Furthermore, one of the mutations in ACTR helix 1 was the preferred Ala → Gly, which reported a Φ_b_ value of 0.24 (Table S1). Thus, overall we deem the input Φ_b_ value data set sound, but as pointed out (23), the number of any single Φ value should be treated with caution and interpreted as weak, intermediate, or strong. Furthermore, several Φ values should preferably be measured for every structural region, which is the case here for helix 1 of ACTR.

**Figure 1.**
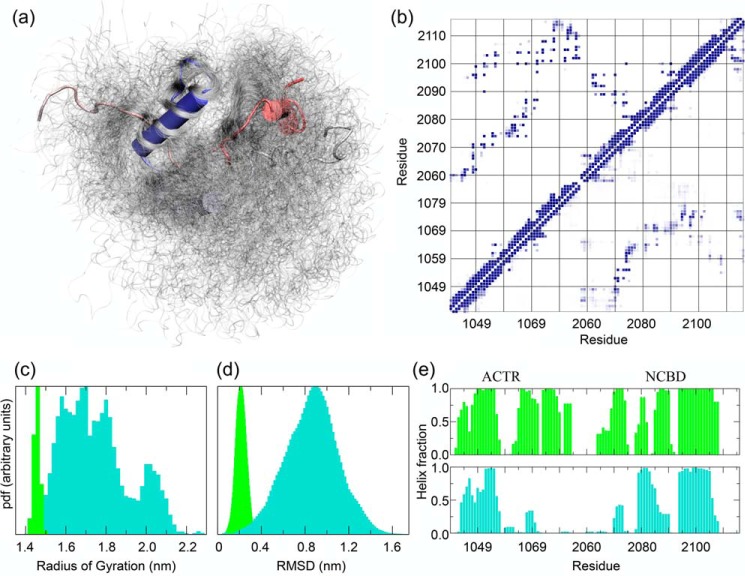
**Model of the transition state ensemble for the binding between ACTR and NCBD.** *a*, structural representation of the TS ensemble with ACTR in *violet* (helix 1) and NCBD in *blue* (helix 3). *b*, contact map analysis for the bound native state (*upper diagonal*) and the TS ensemble (*lower diagonal*). The probability of contact formation goes from white (0) to blue (1). *c* and *d*, comparison of the radius of gyration (*c*) and root mean square deviation distributions (*d*) for the native bound state of ACTR–NCBD (*green bars*) and the TS ensemble (*turquoise bars*). Probability density function (*pdf*) in arbitrary units is shown on the *y* axis. *e*, comparison of the helical content for a simulation of the native bound state of ACTR–NCBD (*green bars*) and the TS ensemble (*turquoise bars*).

Overall, the TS structure is very extended and disordered (Fig. 1). In particular a comparison to a simulation for the ground state of the bimolecular complex highlighted how the TS ensemble is characterized by a highly heterogeneous size (measured by the radius of gyration; Fig. 1*c*), as well as by highly heterogeneous conformers (measured by the distribution of pairwise RMSDs; Fig. 1*d*). Analysis of the secondary structure populations calculated using DSSP (24) and averaged over the ensemble shows that most of the native helical structure in the TS is not formed, except for helix 1 of ACTR (Aα1) and helix 3 of NCBD (Nα3) that are almost native-like (Fig. 1*e*). The tertiary and quaternary structure arrangement as revealed by a contact analysis contains many more weakly populated contacts in the TS than in the native state (Fig. 1*b*). The interface between the two proteins in the TS is diffused and disordered as compared with that of the native complex (Fig. 1*b*), with the TS showing a larger number of less populated contacts (Fig. S1). In the TS the interdomain contacts between the native-like Aα1 and Nα3 are relatively well-formed with respect to other interface contacts (Fig. 1*b*). The overall picture suggests that the two helices are the structural core of the TS around which the remaining elements of the complex organizes to form a very heterogeneous TS structure stabilized by unspecific interactions.

### Testing the binding TS model with mutation

**Figure 2.**
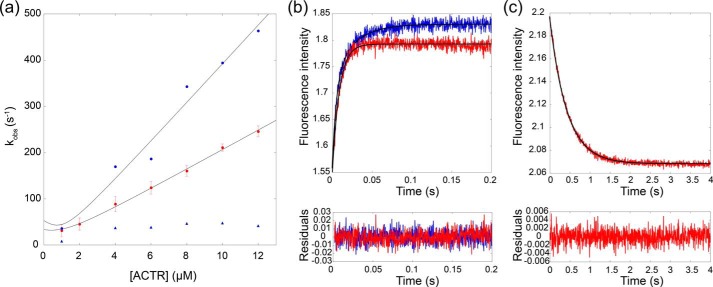
**Binding kinetics for ACTR and NCBD.** *a*, the dependences of *k*_obs_ on ACTR concentration for NCBD_Y2108W_ (pseudo-WT, shown in *red*) and NCBD_T2073A_ (*blue*) at 277 K in 20 mm sodium phosphate (pH 7. 4), 150 mm NaCl. NCBD_T2073A_ exhibited biphasic kinetics where the second phase (*blue triangles*) was rather constant with ACTR concentration. The linear phase was used to calculate the Φ_b_ value. Each *k*_obs_ value of NCBD_Y2108W_ is an average of two different experiments conducted with different samples, and the *error bars* show the standard deviation for *k*_obs_ at each ACTR concentration. *b*, a typical kinetic trace for the binding of NCBD_T2073A_ (*blue*) and NCBD_Y2108W_ (*red*) to ACTR_WT_. The concentrations were 1 μm NCBD and 6 μm ACTR. The *black line* represents the fit to a double exponential function for NCBD_T2073A_ and a single exponential curve for NCBD_Y2108W_. The residuals for the fits are shown below each plot. *c*, the dissociation rate constants were determined in displacement experiments in which NCBD_WT_ was used to displace the different NCBD variants from the complex. The figure shows the displacement of NCBD_Y2108W_ from its complex with ACTR upon addition of NCBD_WT_. The *black line* represents the fit to a single exponential curve, and the residuals are shown below the graph.

To probe the effect of the many weakly populated contacts at the interface between the two proteins, we selected residues involved in such contacts for additional experiments. Based on the contact maps (Fig. 1*b*), six mutants (ACTR_E1065A_, ACTR_E1066A_, ACTR_P1074A_, NCBD_Q2068A_, NCBD_T2073A_, and NCBD_A2098G_) were expressed, purified, and subjected to kinetic binding experiments with pseudo-WT NCBD_Y2108W_ and WT ACTR using the stopped-flow technique (Table 1, Fig. 2, and Figs. S2 and S3) as previously described (16). The same caveats with regard to interpretation of Φ_b_ values, as discussed in the previous section, applies here (23).

**Table 1:** **Experimentally determined binding rate constants, dissociation constants, and Φ_b_ values for the association between ACTR and NCBD** The measurements were conducted in 20 mm sodium phosphate (pH 7.4), 150 mm NaCl at *T* = 277 K unless otherwise stated. Typically, NCBD_Y2108W_ was held constant at 1 μm, and ACTR was added in excess. Association rate constants and *K_d_* values from experiments in which NCBD_Y2108W_ was added in excess over ACTR are shown in parentheses.

ACTR variant	NCBD variant	Interaction probed	*k*_off_	*k*_on_	*K_d_*	Φ_b_
			*s*^−*1*^	μ*m*^−*1*^ *s*^−*1*^	*nm*	
WT	NCBD_Y2108W_		2.2 ± 0.1	21.3 ± 0.4 (17.0 ± 0.6)	103 ± 7 (129 ± 7)	
WT	NCBD_Y2108W_*^a^*		0.49 ± 0.02	95 ± 10	5.2 ± 0.8	
WT	NCBD_Q2068A_	Non-native interactions with the N-terminal of ACTR	2.7 ± 0.1	28.1 ± 0.6	96 ± 6	
WT	NCBD_T2073A_	Non-native interaction with Leu-1052 and residual helical structure	1.08 ± 0.03	41 ± 2	26 ± 2	0.49
WT	NCBD_N2088A_	Cluster of contacts with region 1069–1072 of ACTR	2.3 ± 0.1	22.2 ± 0.8	104 ± 8	
WT	NCBD_A2098G_*^a^*	Formation of Nα3	1.68 ± 0.04	33.8 ± 0.3	50 ± 2	0.45
ACTR_E1065A_	NCBD_Y2108W_	Non-native interaction with backbone of NCBD Lys-2075	2.2 ± 0.1	21.3 ± 0.6 (18 ± 1)	104 ± 8 (122 ± 9)	
ACTR_E1066A_	NCBD_Y2108W_	Non-native interaction with backbone of NCBD Ser-2076	1.73 ± 0.02	22.4 ± 0.3 (17.9 ± 0.7)	77 ± 2 (97 ± 4)	
ACTR_P1074A_	NCBD_Y2108W_	Cluster of contacts with region 2094–2098 of NCBD	1.9 ± 0.1	18.5 ± 0.6 (19.1 ± 0.6)	103 ± 9 (99 ± 6)	

*^a^* The experiments were conducted in 20 mm sodium phosphate (pH 7.4), 150 mm NaCl, 0.7 m TMAO.

NCBD_A2098G_, was used to probe native formation of Nα3 in the TS. The shape of the CD spectrum, which reflects secondary structure content, was slightly different for NCBD_A2098G_ as compared with the other NCBD variants (208 *versus* 222 nm; Fig. S2). This suggests a structural perturbation, and kinetic stopped-flow experiments were therefore conducted in the presence of 0.7 m TMAO, which restored the shape of the CD spectrum for this mutant. The association rate constant for NCBD_A2098G_ decreased 3-fold as compared with the pseudo-WT NCBD_Y2108W_ (in 0.7 m TMAO), whereas the dissociation rate constant increased 3-fold, yielding a Φ_b_ value of 0.45 and thus confirming early formation of native interactions in Nα3. (Φ_b_ in buffer without TMAO was very similar and also in the intermediary category, 0.38.) We note that the binding of the pseudo-WT NCBD_Y2108W_ to WT ACTR in the presence of 0.7 m TMAO exhibited biphasic kinetics, where one phase increased linearly with the concentration of ACTR (the one used to calculate *k*_on_) whereas the second phase had a rather constant *k*_obs_ value of ∼50 s^−1^. The linear kinetic phase reports on the binding event as discussed previously (18) and in the introduction.

The NCBD_T2073A_ mutant, probing both native and non-native intramolecular interactions in the TS, showed a 2-fold increase in association rate constant and a 2-fold decrease in the dissociation rate constant, suggesting a deletion of a nonfavorable interaction in the TS in agreement with the simulation. The results are also consistent with deletion of a native unfavorable interaction between Ile-2089 and the hydroxyl group of Thr-2073 that imposes frustration in the ground states of both free and complexed NCBD, although analysis using the Frustratometer 2 (25) suggests little frustration in this region. In either case, NCBD_T2073A_ yielded a Φ_b_ value of 0.49 compatible with partial native helical population in the TS (Fig. 1*b*). In addition, this mutant exhibited biphasic kinetics with one linear and one apparently constant phase with a *k*_obs_ value of ∼40 s^−1^ in the absence of TMAO, showing that the hydroxyl of the Thr facilitates an intramolecular step on the path to the native bound conformation. The Φ_b_ value calculated from the linear phase reports on the TS for binding as previously discussed (18).

The remaining point mutants (NCBD_Q2068A_, NCBD_N2088A_, ACTR_E1065A_, ACTR_E1066A_, and ACTR_P1074A_) showed no significant effects on the association and dissociation rate constants. For this reason, ΔΔ*G*_Eq_ values were too low (<0.18 kcal mol^−1^) to allow calculation of Φ_b_ values and validation of any specific non-native interactions. However, although not a proof, the lack of effect of the mutations is consistent with an interface between the two proteins in the TS that is malleable, stabilized by unspecific interactions and able to easily rearrange upon mutagenesis.

### Brønsted plots and Φ_b_ values of the ACTR–NCBD interaction

To further assess the role of the respective protein domain in determining the properties of the TS in the coupled binding and folding reaction, we reanalyzed a previous comprehensive data set on the binding kinetics between ACTR and NCBD (26). This data set contains kinetic experiments with pairs of mutations, one in ACTR and one in NCBD (and also including the pseudo-WT variants). Such a double-mutant cycle gives the possibility to calculate interaction (or coupling) free energies ΔΔΔ*G*_C_ between any two residues (27), which was done in the previous study (26). However, these data can also be subjected to Brønsted/Leffler type analysis (28) in which the change in free energy for the TS (ΔΔ*G*_TS_) upon mutational perturbation is plotted *versus* the change in free energy at equilibrium (ΔΔ*G*_Eq_). Such linear free energy relationships were originally devised for physical organic chemistry but adopted by enzymology (29), protein folding (30), and binding (31) studies. We evaluated, first, the slope α of Brønsted plots (ΔΔ*G*_TS_ *versus* ΔΔ*G*_Eq_), which is a measure of the overall resemblance of the TS to the native state (α = 1 for full native interactions and 0 for no native interactions) and, second, Φ_b_ values, which report on local structure. In these data sets, binding kinetics were measured for pseudo-WT NCBD_Y2108W_ and 10 point mutants of NCBD_Y2108W_ *versus* WT ACTR and 8 point mutants of ACTR. For each NCBD variant, 6–8 kinetic experiments with distinct ACTR variants could be performed. Likewise, from the perspective of ACTR, for each ACTR variant 3–10 kinetic experiments with distinct NCBD variants were performed. In total, 87 binding experiments with ACTR and NCBD wildtype and point mutants of 99 possible combinations were included in the analysis. The complexes of some pairs, for example NCBD_I2062V_ and ACTR_L1056A_, were too unstable to allow a kinetic analysis and thus excluded. This data set was previously interpreted in terms of coupling free energies between specific positions in ACTR and NCBD (26).

**Figure 3.**
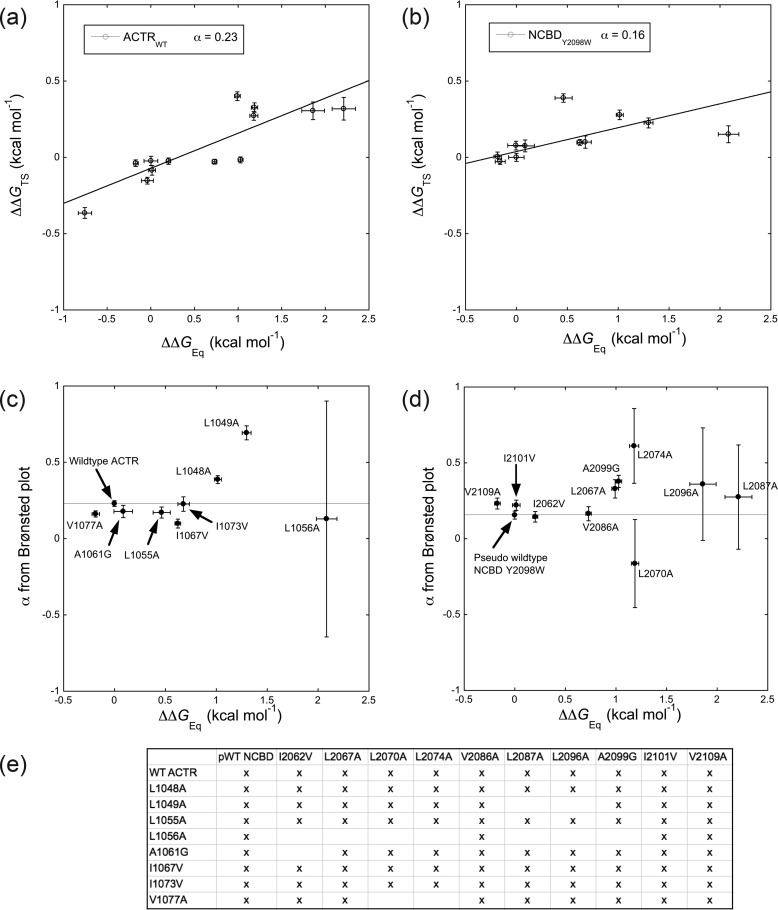
**Linear free energy relationships for the ACTR–NCBD interaction.** *a* and *b*, show two examples of Brønsted plots. Shown are the differences in free energies upon mutation for the binding between ACTR and NCBD (ΔΔ*G*_TS_ plotted *versus* ΔΔ*G*_Eq_) for WT ACTR *versus* a panel of pseudo-WT NCBD_Y2108W_ mutants (*a*) and for pseudo-WT NCBD_Y2108W_ *versus* a panel of ACTR mutants (*b*). A slope (α) close to 0 indicates a low degree of native contacts in the TS, whereas a slope of one suggests that all native contacts are formed in the TS. Moreover, a linear relationship between ΔΔ*G*_TS_ and ΔΔ*G*_Eq_ is indicative of a nucleation–condensation type mechanism where all native contacts form simultaneously around a “nucleus.” On the other hand, nonlinearity is more consistent with intermediates and preformed structure. Similar Brønsted plots were made for each ACTR and pseudo-WT NCBD_Y2108W_ variant. *c* and *d*, the slope α from these Brønsted plots was then plotted against ΔΔ*G*_Eq_ for each variant: ACTR variants (*c*) and pseudo-WT NCBD_Y2108W_ variants (*d*). The *horizontal lines* correspond to the α value of WT ACTR (from *a*, shown in *c*) and pseudo-WT NCBD_Y2108W_ (from *b*, shown in *d*). *e*, all pairs of variants that were included in the analysis are marked by an *X*. The pairs of variants not included were too destabilized by the two mutations, resulting in binding kinetics too fast for the stopped-flow experiments.

The slopes of Brønsted plots were overall similar among NCBD variants (median α = 0.23, average α = 0.24), but with large errors in the slope for NCBD mutations where ΔΔ*G*_Eq_ > 1 kcal mol^−1^ (Fig. 3). A similar pattern is observed for Brønsted plots among ACTR variants (median α = 0.18, average α = 0.25). However, α differs for some point mutants. For NCBD, all four α values, which deviate significantly from that of pseudo-WT NCBD_Y2108W_, were from variants with ΔΔ*G*_Eq_ ≥ 1 kcal mol^−1^ and with slightly perturbed CD spectra (suggesting ground state structural effects on NCBD). Turning to ACTR we observe a similar trend with larger deviation from WT α values when ΔΔ*G*_Eq_ > 1 kcal mol^−1^. The mutated residues of two ACTR variants with higher α values (L1048A and L1049A) cluster in the complex with three NCBD residues giving destabilized NCBD variants (L2067A, L2070A, and L2074A). These residues are part of the α1 helices of ACTR and NCBD, respectively, and could either perturb the folding pathway or, because they are all highly destabilizing and potentially disruptive Leu → Ala mutations, result in structural changes in the ground state complex.

**Figure 4.**
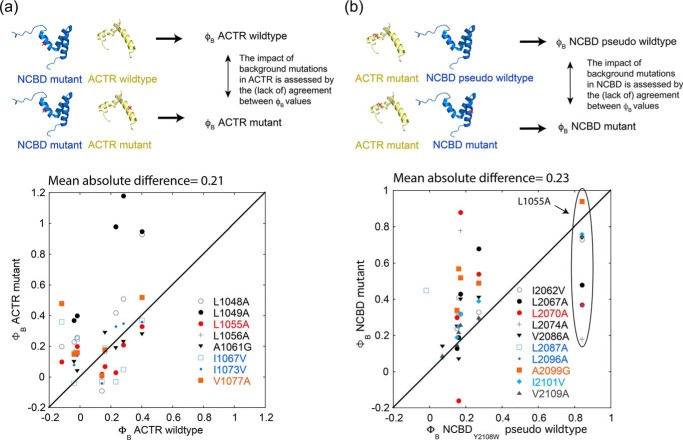
**Φ_b_ values in WT *versus* mutants.** *a*, Φ_b_ values for mutations in NCBD determined for different variants of ACTR. *b*, Φ_b_ values for mutations in ACTR determined for different variants of NCBD. The Leu-1055 mutations are in the *oval*. The *solid line* corresponds to perfect agreement (slope = 1). Some of the Φ_b_ values have very large propagated errors that are not shown for clarity. Φ_b_ values with errors of >1 are not shown. The mean absolute difference between Φ_b_ values in WT and mutant background is shown above each graph. The Protein Data Bank entries 1KBH (11) and 2KKJ (12) were used to draw models of ACTR (*i.e.* hypothetical) and NCBD, respectively.

Second, we calculated Φ_b_ values with each ACTR and NCBD variant as a WT. In this way, we could assess how point mutation in NCBD affected Φ_b_ values for mutations in ACTR and vice versa. Plots of Φ_b_ values for WT ACTR and NCBD, respectively, *versus* Φ_b_ values of their point mutants result in a large scatter. The mean absolute difference between Φ_b_ values in WT and mutant background is ∼0.2 for both NCBD and ACTR variants (Fig. 4). In other words, many of the Φ_b_ values differ between NCBD variants, which means that the mutational perturbation in NCBD affects the measured Φ_b_ value obtained from mutation in ACTR. As visualized by the *error bars* for the slope α from Brønsted plots (Fig. 3, *c* and *d*), Φ_b_ values are more similar for smaller mutational perturbations in NCBD (<1 kcal mol^−1^) in agreement with general guidelines for Φ value analysis in protein folding (23). Considering only ΔΔ*G*_Eq_ < 1 kcal mol^−1^, the Φ_b_–Φ_b_ values show an improved agreement. This is evident from the lower mean absolute difference (0.15 and 0.10 for background mutations in ACTR and NCBD, respectively) between the Φ_b_–Φ_b_ pairs (Fig. S4). Four of the NCBD variants with ΔΔ*G*_Eq_ > 1 have significant ground state effects based on CD. However, Φ for folding were never considered perfectly accurate because of several caveats and should be viewed as low, intermediate, or high. Indeed, the Φ_b_ values in the present study mainly fluctuate within the same category (0–0.3, 0.3–0.6, and 0.6–1).

One of the substitutions, L1049A in helix 1 of ACTR (ΔΔ*G*_Eq_ = 1.3 kcal mol^−1^), was particularly intriguing because all five Φ_b_ values, which could be determined by mutation in NCBD, were significantly higher in the context of ACTR_L1049A_ as compared with ACTR WT (Fig. 4*a*). This is also reflected in the high α = 0.69 ± 0.05. Leu-1049 is situated in the more structured part of the TS (Fig. 1), and destabilization by L1049A might result in a shift of the position of the TS along the reaction coordinate toward a more native conformation in agreement with Hammond behavior as seen in protein folding studies (30). There is a trend to higher Φ_b_ values (Hammond behavior) also for the neighboring amino acid, L1048A (α = 0.39 ± 0.03). Although Φ_b_ values for NCBD correlate well in the backgrounds of ACTR WT and L1055A, Φ_b_ values for Leu-1055 are generally lower with different NCBD variants as compared with pWT NCBD thus displaying anti-Hammond behavior (inside *oval* in Fig. 4*b*). Interestingly, this L1055A mutation is the only one giving a high Φ_b_ value with pWT NCBD. Finally, we note that conservative deletion mutations and in particular Leu → Ala, which removes three methyl groups, will yield Φ_b_ values that report on the tertiary interaction(s) of the side chain but also on the secondary structure, which the residue is part of, because any tertiary interaction will stabilize its associated secondary structure and vice versa.

## Discussion

Coupled binding and folding of IDPs is a complex reaction involving formation of a large number of noncovalent interactions (11, 32, 33). Similarly to folding of protein domains, these reactions can experimentally appear as two state (10, 34) or as in the case of ACTR–NCBD (15, 16) and HPV E7/Rb (35), can involve detectable intermediates. In either case, the fine details of the coupled binding and folding are hard to probe with currently available kinetic instruments. However, by combining experiment with simulation, it is possible to gain a deeper understanding of the molecular events along the binding and folding pathway. We have here used restrained MD simulation in combination with mutagenesis and linear free energy relationships to better understand the binding transition state for the coupled binding and folding of ACTR and NCBD.

IDP interactions studied by Φ_b_ analysis differ in complexity ranging from a single β strand or α helix to several helices (10, 31, 34, 36–41). The ACTR–NCBD interaction involves formation of three helices and a relatively large interface that is topologically complex, resulting in a multistep binding mechanism as reflected in multiple kinetic phases (16, 18, 15). Our present results suggest that the TS for binding is a structurally heterogeneous ensemble of complexes, with a large fraction of weakly populated native and non-native interface contacts, the most native-like regions being helix 1 of ACTR and helix 3 of NCBD. Of notice, a former computational study based on a simplified structure-based model suggested a disordered transition state for the ACTR–NCBD complex associated with an induced-fit mechanisms but could not provide a description of the TS including non-native interactions (42). Mutagenesis based on the structure of the TS indicates that many of these native and non-native interdomain interactions contribute little or nothing in energetic terms to the binding and folding reaction reinforcing a plastic TS. Brønsted plots and Φ_b_ analysis suggests that the total amount of structure in the TS is generally robust, but that specific contacts formed in the TS can change upon mutation of the partner protein. Furthermore, some highly destabilizing mutants suggest that the TS might display Hammond behavior, *i.e.* that the position and structure of the TS approaches the native state when the ACTR–NCBD complex is destabilized by mutation. The Hammond effect is well-established in physical–organic chemistry (43) and has been observed in protein folding (30) but challenged because an apparent TS movement could result from changes in the ground state structures upon mutation (44). In the present case, although the ACTR–NCBD complex tolerates most mutations, some might slightly perturb both the native free and bound states and the TS. Indeed, for a coupled binding and folding reaction with plastic TS and native structures, such movement of TS position and ground state structure appears reasonable and could be a general property whereby IDPs can accommodate mutations more easily than folded proteins (45).

Overall, our data support a model in which the initial events in coupled binding and folding reactions are not following obligatory pathways but involves encounter complexes with large structural variation (46). Thus, most native interactions are formed in an induced fit type mechanism after the rate-limiting barrier as previously suggested for ACTR–NCBD (18, 42, 47), and where intermediates may be detected by kinetics under certain conditions (16). A disordered TS does not require the free states to sample differently ordered “bound-like” states interconverting by extensive conformational changes. Still, such conformational selection may occur in shorter segments like helix 1 for ACTR and helix 3 for NCBD. However, although our kinetic and computational data show that these helices are partially formed in the TS, we cannot resolve the order of events (19), and it is likely that both disordered and ordered conformations of the helices can form the initial encounter complex as suggested for other IDP interactions (32, 48). In a broader context, our data provide support for the emerging view that structural heterogeneity is essential for many protein–protein interactions, with implications for the binding pathway, as well as for the most stable complex (49, 50).

## Experimental procedures

### Determination of the transition state ensembles

Molecular dynamics simulations were performed using the Amber03w force field (51) with the TIP4P/05 water model (52). All the simulations were run in GROMACS (53) using PLUMED2 (54). The van der Waals and Coulomb interactions were implemented with a cutoff at 0.9 nm, and long-range electrostatic effects were treated with the particle mesh Ewald method. Simulations were carried out in the canonical ensemble by thermosetting the system using a stochastic velocity rescaling (55). The starting conformation was taken from an available NMR structure (Protein Data Bank code 1KBH) and modified to account for the Y2108W mutation using SCWRL4 (56). The structure was solvated in ∼7200 water molecules. A standard 298 K simulation, 150 ns long, was performed as a reference for the native state ensemble.

Binding TS ensembles were determined following a previously described procedure based on the interpretation of Φ value analysis in terms of fraction of native contacts (20–22). Given a set of experimental Φ values, a pseudo energy term is added to a force field as the squared difference between experimental and simulated Φ values. This restraint provides an effective potential that can in principle account for multiple effects like the ionic strength. The Φ value for a residue is calculated from the fraction of native contacts that it makes in a conformation. Given two residues that are not nearest neighbors, the native contacts between them are defined as the number of heavy side-chain atoms within 0.65 nm in the native structure. With this approach only Φ values between 0 and 1 can be incorporated as structural restraints. The underlying hypothesis is that the average structure satisfying all Φ values can be a good representation, at least at the residue-level resolution, of the TS. This hypothesis, even if not necessarily true, has previously proved powerful by providing testable predictions (22, 57–60). The TS ensemble was generated using simulated annealing, 1340 annealing cycles, 150 ps long, in which the temperature is varied between 298 and 383 K and performed for a nominal simulation time of 200 ns. Only the structures sampled at the reference temperature for the latter 1000 cycles are retained for further analysis, resulting in a TS ensemble of 1000 structures. The sampling of the conformational space is robust given its heterogeneity. The pair-wise RMSD support the notion that a number of uncorrelated structures are sampled. Furthermore, a cluster analysis on the first 500 structures or including all the structures shows a converged number of clusters (Fig. S5).

### Protein expression and purification

Site-directed mutants of ACTR and the pseudo-WT NCBD_Y2108W_ were generated by whole plasmid mutagenesis (QuikChange), using a complementary primer pair with basepair mismatches at the mutation site and flanked by 15 bp on each side. Protein expression and purification were performed as previously described (16). Protein concentrations were determined by absorbance at 280 nm for protein variants that contained Tyr or Trp residues. To determine the concentration of the ACTR variants, which are lacking aromatic residues, absorbance at 205 nm was measured, and an extinction coefficient of 250,000 cm^−1^ m^−1^ was used to estimate the protein concentration.

### Kinetic experiments

The kinetic experiments were conducted on an SX-17MV stopped-flow spectrophotometer. The excitation wavelength was set to 280 nm and a 320-nm-long pass filter was used to detect the emitted light. All experiments were performed at 277 K in 20 mm sodium phosphate (pH 7.4), 150 mm NaCl with or without 0.7 m TMAO. It has been shown previously that NCBD remains monomeric in presence of TMAO (12). Typically, the binding kinetics were measured using 1 μm of the NCBD_Y2108W_ pseudo-WT and varying the concentration of ACTR between 1 and 12 μm. In addition, the binding kinetics of the ACTR mutants were measured using 1 μm of the respective ACTR mutant and varying concentrations of the NCBD_Y2108W_ pseudo-WT. The kinetic traces (each trace typically an average of three traces) were fitted to either a single exponential or double exponential function using the Applied Photophysics software to obtain the observed rate constant (*k*_obs_) at each ACTR or NCBD concentration. The resulting plots of *k*_obs_ values against different ACTR or NCBD concentrations were fitted using KaleidaGraph *versus* 4.5 (Synergy Software) to the solution for reversible bimolecular binding to obtain the association rate constant (*k*_on_) for each variant (61). The dissociation rate constants (*k*_off_) were measured in displacement experiments, where WT NCBD was used to displace the different NCBD variants from the complexes. The concentration of proteins in the complex was 1 μm NCBD and 1 μm ACTR. At high concentrations of WT NCBD, *k*_obs_ reaches a plateau, and the mean *k*_obs_ value from the plateau region is approximated as *k*_off_. The standard deviation of the average from these points is taken as the error for *k*_off_. All kinetic data are provided as a supporting Excel file.

### CD spectroscopy

CD spectra were measured on a JASCO J-1500 CD spectrophotometer between 200 and 260 nm. The buffer conditions were 20 mm NaP_i_ (pH 7.4), 150 mm NaCl with or without 0.7 m TMAO, and the temperature for all measurements was 277 K. The bandwidth was set to 0.1 nm, scanning speed was 50 nm/min, and data points were sampled every 0.1 nm. Each spectrum is an average of three accumulations.

## Author contributions

E. K., P. J., and C. C. data curation; E. K., J. D., S. G., P. J., and C. C. formal analysis; E. K., E. A., and C. C. investigation; E. K., P. J., and C. C. visualization; E. K., E. A., and C. C. methodology; E. K., P. J., and C. C. writing-original draft; E. K., J. D., S. G., P. J., and C. C. writing-review and editing; S. G., P. J., and C. C. conceptualization; P. J. and C. C. resources; P. J. supervision; P. J. funding acquisition; P. J. project administration.

## Supplementary Material

Supporting Information

## Footnotes

3 The abbreviations used are:

IDP: intrinsically disordered protein
ACTR: activation domain from the p160 transcriptional co-activator for thyroid hormone and retinoid receptors
NCBD: nuclear co-activator binding domain
TS: transition state
TMAO: trimethylamine *N*-oxide
MD: molecular dynamics.
