# Supplementary material for: A structurally heterogeneous transition state underlies coupled binding and folding of disordered proteins
Source: J Biol Chem. 2018 Dec 4;294(4):1230–9. doi: 10.1074/jbc.RA118.005854 (PMC6349112; doi:10.1074/jbc.RA118.005854)
Supplement: Supporting Information [file supp_294_4_1230__index.html]

A structurally heterogeneous transition state underlies coupled binding and folding of disordered proteins — A heterogeneous transition state for an IDP interaction — A structurally heterogeneous transition state underlies coupled binding and folding of disordered proteins — A heterogeneous transition state for an IDP interaction — Supporting Information 

# A structurally heterogeneous transition state underlies coupled binding and folding of disordered proteins

## Supporting Information

- Supporting Information (to be published online) - All supporting information contained in one word file. Supporting figures are also provided separately. I wasn't sure if you want to format it before publishing but in the end it should be a pdf file.
- Supporting Information (to be published online) - Excel file containing experimental kinetic parameters and calculated phi\_b values
