# Supplementary material for: A structurally heterogeneous transition state underlies coupled binding and folding of disordered proteins
Source: J Biol Chem. 2018 Dec 4;294(4):1230–9. doi: 10.1074/jbc.RA118.005854 (PMC6349112; doi:10.1074/jbc.RA118.005854)
Supplement: Supporting Information [file supp_RA118.005854_140775_2_supp_243885_pj0xsk.docx]

**SUPPORTING INFORMATION**

A structurally heterogeneous transition state for coupled binding and folding of disordered proteins

Elin Karlsson^1^, Eva Andersson^1^, Jakob Dogan^2^, Stefano Gianni^3^, Per Jemth^1,^*, and Carlo Camilloni^4,^*

^1^Department of Medical Biochemistry and Microbiology, Uppsala University, BMC Box 582, SE-75123 Uppsala, Sweden.

^2^Department of Biochemistry and Biophysics, Stockholm University, SE-10691 Stockholm, Sweden

^3^Istituto Pasteur-Fondazione Cenci Bolognetti and Istituto di Biologia e Patologia Molecolari del CNR, Dipartimento di Scienze Biochimiche “A. Rossi Fanelli,” Sapienza Università di Roma, 00185 Rome, Italy.

^4^Dipartimento di Bioscienze, Università degli Studi di Milano, 20133 Milano, Italy

Running title: A heterogeneous transition state for an IDP interaction

*Correspondence to

Carlo Camilloni, [carlo.camilloni@unimi.it](mailto:carlo.camilloni@unimi.it), phone: +39- 02-503-14918‬

Per Jemth, [Per.Jemth@imbim.uu.se](mailto:Per.Jemth@imbim.uu.se), phone: +46-18-471 4557

**Supplementary Figure S1. Analysis of the ACTR/NCBD interface.** On the left is a comparison of the average population of interface contacts in the ground state (green) and TS ensemble (turquoise). This is calculated only for contacts at least 5% populated. On the right is a comparison of the number of interface contacts at least 5% populated.

**Supplementary Figure S2.** **Experimental circular dichroism data for each mutant included in the study.** Far-UV CD spectra of the ACTR variants (left) and the NCBD variants (right) used in the experiments. The measurements were conducted in 20 mM sodium phosphate (pH 7.4), 150 mM NaCl and at T= 277 K. NCBD_A2098G_* was measured in the presence of 0.7 M TMAO.

**Supplementary Figure S3.** **Experimental data from stopped flow experiments for each mutant included in the study compared to the wildtype (NCBD_Y2108W_ and ACTR_WT_, the solid best fit curve is shown in all panels).** The experiments were conducted in 20 mM NaPi (pH 7.4), 150 mM NaCl at T= 277 K unless otherwise stated. Pseudo-wildtype NCBD in the presence of 0.7 M TMAO and the NCBD_T2073A_ mutant exhibited biphasic kinetics. NCBD_Y2108W_ and NCBD_Q2068A_ were measured twice and the mean k_obs_ values were calculated. The error bars show the standard deviation for the replicate experiments. The experiments labeled with * were conducted in 20 mM sodium phosphate (pH 7.4), 150 mM NaCl, 0.7 M TMAO.

**Supplementary Figure S4.** **Φ_b_ values in wildtype versus mutants** **where ΔΔG < 1 kcal mol^-1^ for the complex.** (a) Φ_b_ values for mutations in NCBD with ACTR variants as background. (b) Φ_b_ values for mutations in ACTR with NCBD variants as background. The solid line corresponds to perfect agreement (slope = 1). The mean absolute difference between Φ_b_ values for mutants and wildtype is shown for each graph.


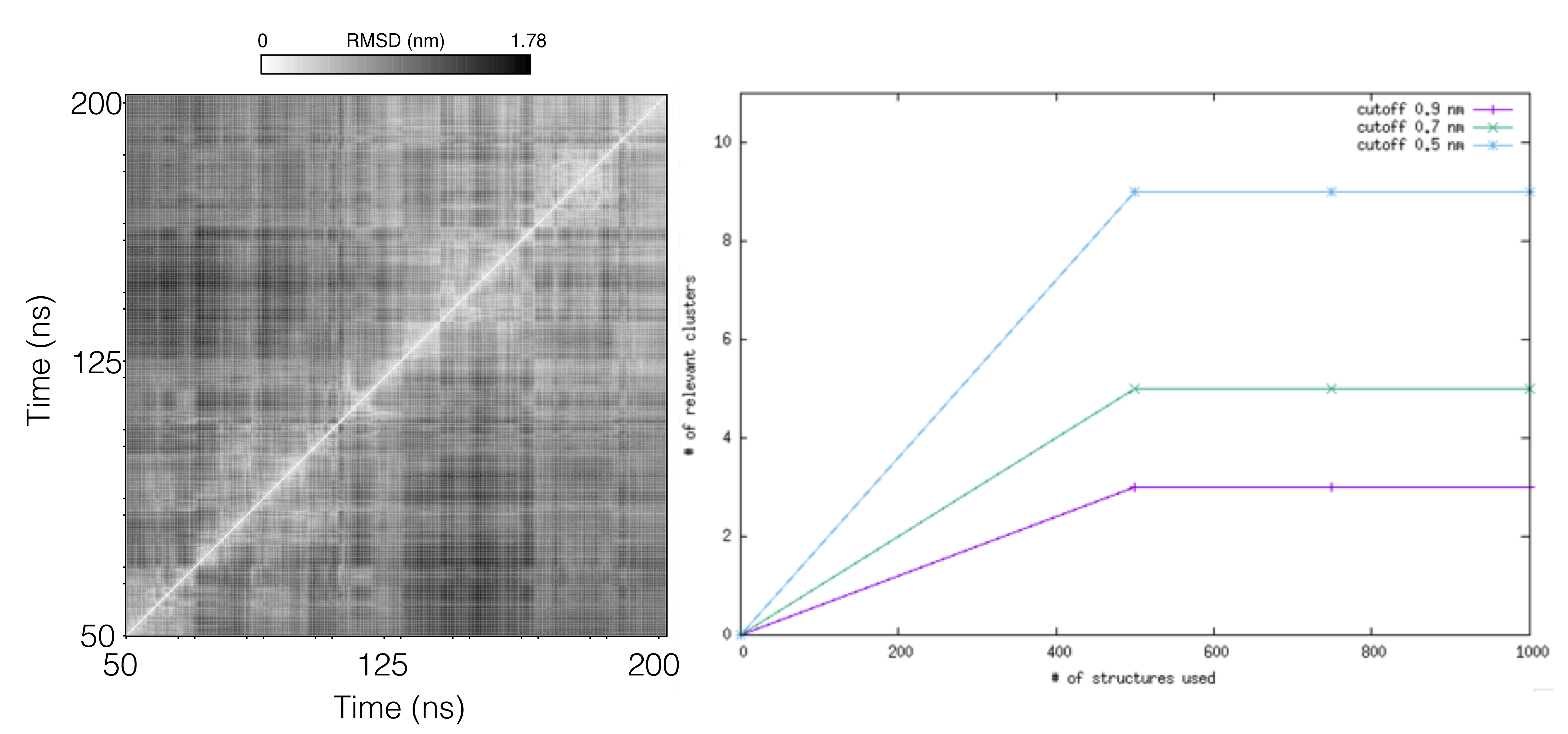


**Supplementary Figure S5. Sampling quality for the TS ensemble simulation.** On the left is shown the pairwise RMSD among all the conformations of the ensemble. This shows the overall independence of the observed structures. On the right is shown the number of relevant populated clusters (>5%) as a function of the number of structures employed. This suggest that already the first half of the simulation was able to capture the relevant conformational space. The clustering is robust with respect to the choice of the cut-off parameter.

**Supplementary Table 1**

Mutations and the corresponding Φ_b_ values used as restraints in the MD simulation.

| Residue  number in pdb file 1KBH | Mutation and associated domain. (Residue number for full length protein) | | ΔΔG_eq_  kcal mol^-1^ | Error  ΔΔG_eq_  kcal mol^-1^ | Φ_b_ | Error  Φ_b_ |
| --- | --- | --- | --- | --- | --- | --- |
| 4 | S1043M | ACTR | -0.19 | 0.02 | 0.66 | 0.09 |
| 8 | A1047G | ACTR | 0.29 | 0.02 | 0.24 | 0.05 |
| 9 | L1048A | ACTR | 1.02 | 0.02 | 0.27 | 0.02 |
| 10 | L1049A | ACTR | 1.30 | 0.02 | 0.17 | 0.02 |
| 11 | D1050E | ACTR | -0.21 | 0.02 | 0.35 | 0.07 |
| 15 | T1054Q | ACTR | -0.18 | 0.02 | 0.74 | 0.12 |
| 16 | L1055A | ACTR | 0.46 | 0.05 | 0.85 | 0.10 |
| 17 | L1056A | ACTR | 2.09 | 0.06 | 0.07 | 0.02 |
| 25 | L1064A | ACTR | 2.54 | 0.13 | 0.06 | 0.02 |
| 28 | I1067V | ACTR | 0.62 | 0.02 | 0.16 | 0.02 |
| 32 | L1071A | ACTR | 3.14 | 0.16 | 0.14 | 0.03 |
| 34 | I1073V | ACTR | 0.68 | 0.03 | 0.15 | 0.05 |
| 38 | V1077A | ACTR | -0.19 | 0.02 | 0.00 | 0.13 |
| 57 | L2067A | NCBD | 0.63 | 0.08 | 0.00 | 0.09 |
| 60 | L2070A | NCBD | 1.21 | 0.07 | 0.20 | 0.04 |
| 64 | L2074A | NCBD | 1.26 | 0.07 | 0.19 | 0.03 |
| 76 | V2086A | NCBD | 0.73 | 0.02 | 0.00 | 0.02 |
| 77 | L2087A | NCBD | 2.21 | 0.09 | 0.14 | 0.03 |
| 86 | L2096A | NCBD | 1.86 | 0.09 | 0.16 | 0.03 |
| 89 | A2099G | NCBD | 1.18 | 0.07 | 0.23 | 0.04 |
| 99 | V2109A | NCBD | -0.17 | 0.02 | 0.22 | 0.09 |
